# DEBFold: Computational Identification of RNA Secondary Structures for Sequences across Structural Families Using Deep Learning

**DOI:** 10.1021/acs.jcim.4c00458

**Published:** 2024-04-22

**Authors:** Tzu-Hsien Yang

**Affiliations:** † Department of Biomedical Engineering, National Cheng Kung University, No.1, University Road, Tainan 701, Taiwan; ‡ Medical Device Innovation Center, National Cheng Kung University, No.1, University Road, Tainan 701, Taiwan

## Abstract

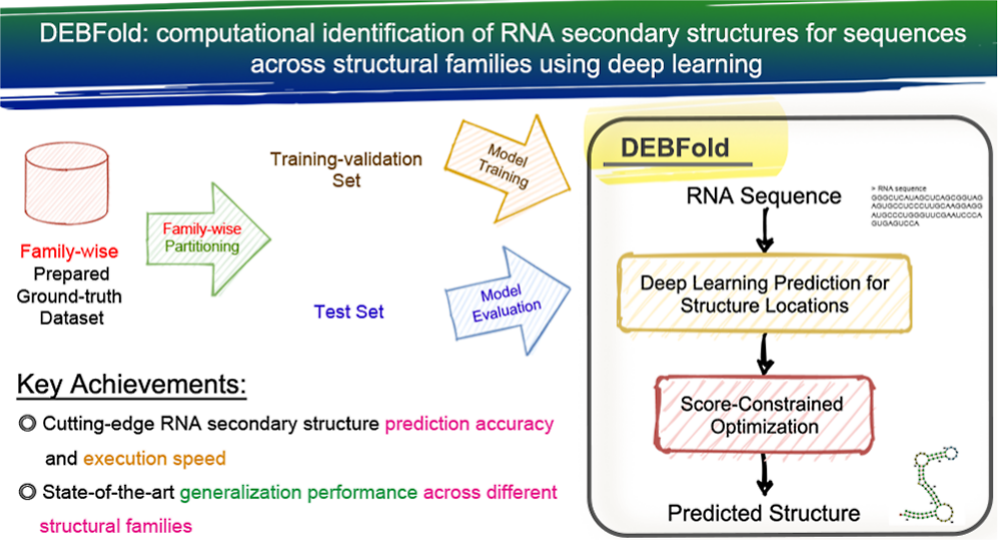

It is now known that RNAs play more active roles in cellular pathways beyond simply serving as transcription templates. These biological mechanisms might be mediated by higher RNA stereo conformations, triggering the need to understand RNA secondary structures first. However, experimental protocols for solving RNA structures are unavailable for large-scale investigation due to their high costs and time-consuming nature. Various computational tools were thus developed to predict the RNA secondary structures from sequences. Recently, deep networks have been investigated to help predict RNA structures directly from their sequences. However, existing deep-learning-based tools are more or less suffering from model overfitting due to their complicated problem formulation and defective model training processes, limiting their applications across sequences from different structural families. In this research, we designed a two-stage RNA structure prediction strategy called DEBFold (deep ensemble boosting and folding) based on convolution encoding/decoding and self-attention mechanisms to enhance the existing thermodynamic structure models. Moreover, the model training process followed rigorous steps to achieve an acceptable prediction generalization. On the family-wise reserved test sets and the PDB-derived test set, DEBFold achieves better structure prediction performance over traditional tools and existing deep-learning methods. In summary, we obtained a cutting-edge deep-learning-based structure prediction tool with supreme across-family generalization performance. The DEBFold tool can be accessed at https://cobis.bme.ncku.edu.tw/DEBFold/.

## Introduction

RNA (ribonucleic acid) molecules in cells can serve as not only transcription templates but also noncoding RNAs.^1^ Many of them carry out specific cellular functions by using their sequence patterns or folding structures.^2,3^ For example, internal ribosome entry site elements can regulate the translation initiation of transcripts under stress conditions via their nonconservative sequence patterns and partial secondary structures.^4,5^ Since RNA secondary structures can form the subdomains in tertiary structures^6^ and can carry out critical cellular functions by themselves,^7^ figuring out the potential secondary structures of newly found RNA sequences becomes routine in RNA molecule studies.^8^

Experimental approaches for solving RNA structures include X-ray crystallography, nuclear magnetic resonance (NMR), and cryo-electron microscopy.^9^ These experiments can recognize the intramolecular RNA base–pair interactions within several angstroms.^10^ However, a long period of technician training time and high costs are required to carry out these experimental approaches. Recently, several chemical probing methods were developed, including the dimethyl sulfate (DMS) and the selective 2′-hydroxyl analyzed by primer extension (SHAPE) reagents treatment, to help identify the existence of structure pairings on sequence locations by detecting reverse transcriptase-stopping sites.^1^ When coupled with next-generation sequencing techniques, genome-wide structural information can be obtained.^11^ Nevertheless, these chemical probing approaches indicate only the existence of some structural forms. They also require additional folding prediction to provide RNA structure–function insights. Because of these reasons, in silico algorithms for identifying RNA structures are essential in the large-scale study of RNA sequences.

Traditional knowledge-based RNA secondary structure prediction algorithms utilize two approaches. The first type of algorithm assumes that an RNA sequence folds into the structure with the minimum free energy. The structures occupying the least free energy states are found through dynamic programming^12^ or Boltzmann partition functions.^13^ Researchers also developed a second type of algorithm that further incorporates multiple sequence alignment results when finding the minimum free energy structures.^14^ This type of tool sifts out the consensus structure either by simultaneous folding and aligning or aligning before folding.^15^ Recently, with the development of chemical structure probing reagents, many tools can also consider probing read scores as soft constraints to enhance structure prediction performance.^16^ Although a bundle of tools based on these two approaches has been proposed, these tools bear drawbacks that deteriorate prediction accuracy. First, free energy minimization algorithms may suffer from incomplete parametrization of the thermal models, triggering lower sensitivity.^7,17,18^ Second, a well-chosen homologous sequence family is indispensable for homology alignment-based tools.^19^ This requirement is usually not feasible for large-scale RNA profiling. Because of the aforementioned issues, researchers have tried to adopt data-oriented methods based on machine learning and deep-learning techniques as another way to solve the RNA structure prediction problem.

Early machine learning-based methods for RNA secondary structure prediction assisted the prediction process by refitting some predefined parameters in the thermal models.^20^ Later, some of these data-driven methods further tried to deal with the prediction problem directly from the sequence level.^21,22^ As a further step from feature-based algorithms, deep-learning tools have become promising for solving RNA structures in an end-to-end manner. However, existing deep-learning-based tools formulated the structure representation using 2D matrix encoding. Since there are far more unpaired position tuples, the 2D matrix structure encoding can easily result in unbalanced positive and negative targets, leading to the need for a large diversity in the training data sets. In a recent study, Szikszai et al.^23^ found that structure diversities within the data set are fundamental for training structure prediction models. They pinpointed that existing tools were trained only on data with sequence base variations, which suggested the occurrence of model overfitting. Because of the high parametrization and defect training processes, no robust result for diverse RNA structures was ready in deep-learning-based tools.^23^

In this research, we devised a two-stage strategy called DEBFold (deep ensemble boosting and folding) to overcome the prevalent overfitting issue in deep-learning-based structure prediction tools. Instead of assuming a sparse 2D structure matrix formulation, DEBFold is based on a more compact 1D linear representation and was trained with a family-wise prepared ground-truth data set. We arranged deep convolution encoding/decoding and self-attention mechanisms to integrate and boost existing thermal structure models in DEBFold for better RNA structure prediction. Compared with traditional knowledge-based or deep-learning-based structure prediction methods, DEBFold achieved better F1 score performance on the reserved structure-independent test sets and the PDB-derived test set, indicating good generalization across RNA structural families. Moreover, we demonstrated that DEBFold is robust in its pipeline design. These results suggest that DEBFold is the state-of-the-art deep-learning-based tool that handles cross-family RNA structure prediction. Finally, we implemented a web interface (https://cobis.bme.ncku.edu.tw/DEBFold/) that is freely available for researchers to facilitate the usage of DEBFold.

## Methods and Data Sets

### DEBFold Workflow

**Figure 1:**
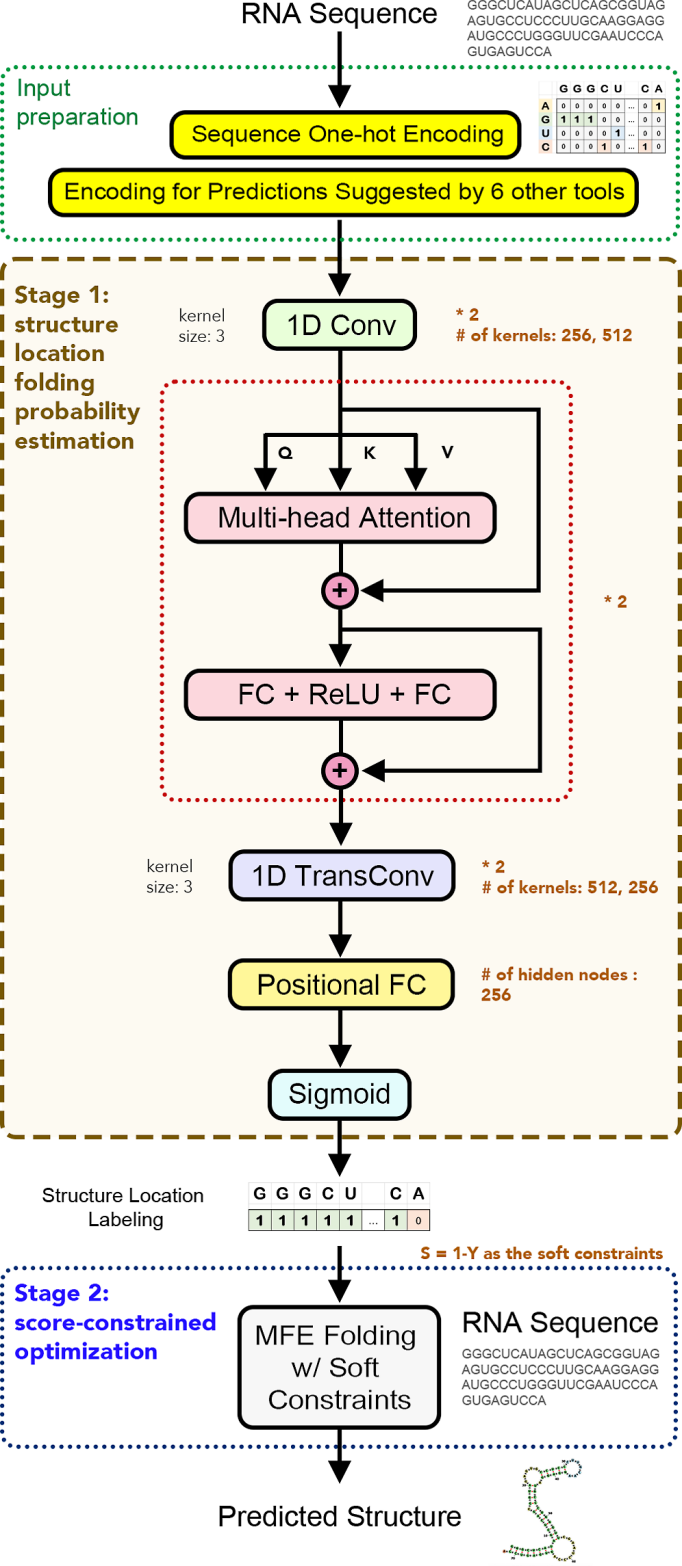
Overview of DEBFold. The example RNA structure is visualized by forna.^24^ Notations: Conv—convolution layers; FC—fully connected layers; TransConv—transpose convolution layers; MFE: minimum free energy.

In this research, we designed a deep-learning workflow that integrates knowledge of different thermodynamic structure models with constrained optimization to tackle the RNA secondary structure folding problem. The designed integration algorithm is called DEBFold (deep ensemble boosting and folding). The DEBFold workflow comprises two stages to convert the given RNA sequences into their potential secondary structures (Figure 1): (1) structure location folding probability estimation. DEBFold was designed to take an RNA sequence into a deep-learning network that helps to integrate knowledge from various thermal models. Then, it generates the folding constraints similar to the SHAPE experiment results. (2) SHAPE-like constrained optimization. In the second stage, free energy minimization constrained by the predicted structure location folding probability will help provide the final structure prediction. These two stages are elaborated on in the following subsections.

#### Stage 1: Structure Location Folding Probability Estimation

In Stage 1, DEBFold tries to identify the potential locations that are involved in the final structure pairings based on a structure location identification deep network. DEBFold takes an RNA sequence as the single input. Before the deep network, the pipeline prepares the network input tensor based on the given sequence. The sequence of the given RNA is one-hot encoded into an *l* x 4 tensor, where *l* is the length of the sequence. Then, to integrate existing thermodynamics structure models, DEBFold collects the prediction results suggested by 6 different single-sequence prediction tools. The prediction results from the following tools were selected to be part of the DEBFold-prepared input tensors: RNAfold,^25^ IPknot,^6^ MaxExpect,^26^ ProbKnot,^27^ RNAprob,^28^ and Fold.^29^ These single-sequence input structure prediction tools were selected based on their prediction accuracy, calculation efficiency, and ease of incorporation. In our previous research,^3^ it was evaluated that RNAfold,^25^ IPknot,^6^ MaxExpect,^26^ and ProbKnot^27^ achieved acceptable structure prediction performance within acceptable time requirements. We further included RNAprob^28^ and Fold^29^ to diversify the thermal models while still limiting the time required to generate these inputs. Although ShapeKnots and HotKnots can provide additional thermal model diversity, these two tools have a much longer processing time for long sequences and thus are not integrated. The prediction results of the 6 tools under consideration are separately encoded into individual tensor *P*_*i*_, where *P*_*ij*_ = 1 if the *j*th location is said to be paired in the predicted structure of tool *i*. At the end of this step, the sequence one-hot encoding and the *P*_*i*_’s are stacked into one *l* × 10 tensor. Since GPU parallel computation requires the lengths of the training batch tensors to be the same, we zero-padded the length of the network input tensor to 512 during the cross-validation training process. For prediction, this model does not need this padding. Therefore, variable-length RNAs can be fed into the structure location identification network when used in prediction. However, because the ground-truth data set collected in this research mainly consists of sequences shorter than 512 bps, the most suitable value of *l* is suggested to be less than 512 nt long.

The structure location identification deep network can be divided into 3 parts: feature extraction, self-attention, and structure location folding probability formation. The first part of the deep network mixes the input information to extract valuable features for the structure location identification. In previous research, convolution operations have been successfully used to extract sequence pattern features.^30,31^ Therefore, consecutive convolution operations (eqs 1 and 2) were adopted in the deep network of DEBFold for extracting sequence and structure features
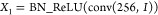
1
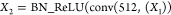
2where *I* is the *l* × 10 input tensor and BN_ReLU refers to the operation of ReLU activation, followed by batch normalization. In the above equation, the convolution operation conv(*n*,*T*) with *n* 1D kernels of size 3 on the *l* × *c* tensor *T* (using stride 2) is defined as previously suggested^32,33^ (eq 3)
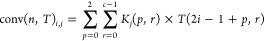
3where *T*(−1) is the zero-padded element, , *j* is in the range of 0 and (*n* – 1), and *K*_*j*_ is the *j*th kernel.

After the features centered at each location are extracted (as *X*_2_ in eq 2), the mutual interactions between each location are considered by the self-attention operation (eqs 4 and 6). A self-attention block helps compute the mutual influence between two nucleotide locations within the given sequence. In order to avoid the gradient vanishing problem in deep neural networks,^34^ a residual architecture was also implemented in the self-attention block (eqs 5 and 7)
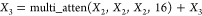
4
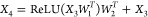
5
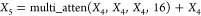
6
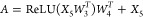
7where (*W*_1_, *W*_2_) and (*W*_3_, *W*_4_) are the trainable weights with internal positional feed-forward dimension 1024, and multi_atten(.) is the multihead attention operation. In DEBFold, the self-attention operation was repeated twice in the network architecture. The multihead attention module, denoted as multi_atten(*Q*, *K*, *V*, *n*), is defined as the following^35^
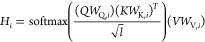
8
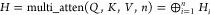
9where *W*_Q,*i*_/*W*_K,*i*_/*W*_V,*i*_ are the query/key/value weight matrices that map Q, K, and V into tensors of dimension 512, *l* is the length of the Q/K/V tensors, *n* is the number of heads used in this operation, ⊕ represents the tensor concatenation operation, and *H* is the computed multihead attention tensor. The self-attention operation is obtained by setting the *K*, *Q*, and *V* tensors to be identical. The number of heads in this network was picked to be 16. After the self-attention block, the sequence features for each location are weighted by the potential influences among different locations.

In the last step of this stage, the locations involved in the final structure pairings are identified in the form of potential pairing probabilities based on the final attended hidden feature tensor *A* (from eq 7). Since the final structure locations are marked segments along the sequence position, DEBFold resorts to consecutive 1D transpose convolutions (eqs 10 and 11. See eqs 12–14 for its definition) to help decode *A* (from eq 7) to produce segment marking along the positions
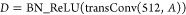
10
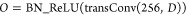
11

In the above equations, the 1D transpose convolution transConv(*n*, *F*) using *n* kernels of size 3 on the *l* × *c* tensor *F* (with stride 2) is calculated based on the following formula
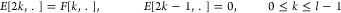
12
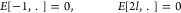
13
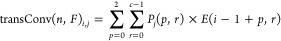
14where *E* is the extension tensor for *F*, 0 ≤ *i* ≤ 2*l* – 1, *j* is in the range of 0 and (*n* – 1), and *P*_*j*_ is the *j* kernel of the transpose convolution operation. Finally, the structure location folding probability of each location *i* in the output tensor *Y* is individually computed based on *O* (from eq 11) as
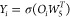
where *W*_5_ is the 256 × 1 trainable weight matrix shared among all base locations and σ is the sigmoid function. The choice of a weight matrix of the same size as the internal hidden dimension fulfills the need to accept RNAs with variant lengths, making DEBFold unlimited by RNA sequence lengths.

#### Stage 2: Score-Constrained Optimization Folding

It has been shown that structure probing values as soft folding constraints can benefit the overall RNA secondary structure prediction.^36^ DEBFold utilizes this concept and incorporates a two-stage pipeline to predict the RNA secondary structures. In Stage 1 of DEBFold, we obtain a structure location folding probability tensor, indicating the possibility of each location being involved in the final structure. The probabilities are inversely related to the read scores measured in probing experiments such as SHAPE-seq. Hence, we defined the SHAPE-like score tensor *S* by *S* = 1 – *Y*. The computed score tensor *S* is then provided as the SHAPE score file option for the minimum free energy structure prediction optimization tools to be used in soft constraint calculation. In DEBFold, we adopted the linear equation proposed by Deigan et al.^37^ and used the default slope and intercept values in each tool for this equation to convert the SHAPE-like scores into pseudoenergy terms in the optimization process. With the guidance of score tensor *S*, more accurate RNA structure predictions can be obtained in the constrained optimization. In the final design of DEBFold, the Fold algorithm^29^ is chosen as the default constrained optimization algorithm to be coupled with the score tensor *S* for generating the final structure prediction.

#### Model Training Hyperparameters

DEBFold Stage 1 is formed by a deep neural network, and we utilized cross-validation (CV) and learning-curve techniques to select the proper hyperparameters. The sum of the binary cross-entropy for each base location was selected as the overall loss function to optimize the deep network. Since the available structural families are not abundant, 25-CV was adopted to fully utilize the data set. In order to avoid overfitting, the average training and validation learning curves were monitored based on the partial loss and partial F1 scores, which exclude the loss and F1 calculation of the padded locations after the original sequence length in each RNA. The final hyperparameters chosen in DEBFold are summarized as follows: (1) training epochs: 250; (2) batch training size: 512; (3) the optimization update algorithm: Adam; (4) learning rate and decay schedule: cosine decay (minimum learning rate = 1 × 10^–13^) in the first 227 epochs and then exponential decay (multiplicative factor 1 × 10^–10^) for the rest of the 23 epochs; (5) dropout layers with dropout rate = 0.1 and 0.4 were added to the residual layers and convolution/transpose convolution layers, respectively, to regularize the deep network; and (6) positive weights = 1.8 in the binary cross-entropy loss. The training process was carried out using a NVIDIA RTX 4080 GPU.

### Family-Wise Processed RNA Structure Ground-Truth Data Set

Model training in data-based learning approaches requires the data diversity in the training set to mimic the degree of variations in real problems.^38^ Researchers have experimented that a large number of sequences from a small number of structural families can easily lead to model overfitting.^23^ The possible reason for the situation is that structurally similar sequences provide limited variations for deep models to fetch the inherent patterns that map sequences to the corresponding structures. In order to prepare a ground-truth data set that incorporates a high structure diversity, we downloaded the data deposited in bpRNA-1m^39^ and retrieved the sequences with known Rfam families. In bpRNA-1m, RNAs curated from Rfam 12.2^40^ were selected and processed. In total, 43,269 valid RNA sequences from 2125 families were found.

In order to tune and evaluate DEBFold in a structural family independent manner, the ground-truth data set is divided and sampled into two parts: the training-validation and test sets. Since RNAs in the same family are structurally similar,^23^ the data processing was carried out in a family-wise manner. We resorted to 25-fold cross-validation in DEBFold Stage 1 model training due to the scarcity of known distinct RNA structural families in the collected ground-truth data set. However, the average of the cross-validation results provides only an optimistic estimation of the model performance. An extra test set should be set aside as totally clean data for a fair performance evaluation. Therefore, among the gathered 2125 families, 44 (2%, or 1/50 due to 25-fold cross-validation, of the total 2125 families) of them were reserved in the test set. These 44 families were selected by stratified sampling that preserves the length distribution of all 2125 RNA families. In the calculation of the length distribution of RNA families, the longest sequence within a structural family was chosen as the representative. These 44 families were also ensured to be in distinct Rfam clans from the rest of the remaining families. The remaining 2081 families were used as the training-validation folds in cross-validation. We sampled at most 5 sequences from the 2081 families to be included in the training-validation set (8782 sequences in total). Finally, we took one sequence from each of the 44 test families for testing, resulting in a test set consisting of 44 RNAs. This test set is termed TestSetα in this research. We further confirmed the sequence similarity between the training-validation and test RNA sequences using CD-HIT.^41^ Under the threshold of 0.85, TestSetα contains no RNA with sequence similarity to any member of the training-validation set. In order to have more extensive testing, we further prepared two additional independent test sets (TestSetβ and TestSetγ) as described in the following two subsections.

#### Contamination-Free Family-Wise Independent Test Set for Evaluating Existing Deep-Learning-Based Structure Prediction Tools

In this research, we also tried to compare the performance of DEBFold with existing deep-learning-based structure prediction tools. While SPOT-RNA and SPOT-RNA2 were originally trained on the bpRNA-1m data set and there is no retraining code for these tools, it is necessary to gather some other independent test set than TestSetα, which was derived from bpRNA-1m, for ensuring a fair generalization evaluation.^38^ For this purpose, we downloaded the bpRNA-new data set from the MXfold2 work^22^ and prepared an independent test set for fairly evaluating the performance of DEBFold with existing deep-learning-based prediction tools. The so-called bpRNA-new data set from the MXfold2 work includes RNA sequences from Rfam 14.4 but excluded those already in Rfam 12.2. We further reorganized the bpRNA-new data set to better help evaluate the deep-learning model performance. In the downloaded bpRNA-new data set, there are a total of 489 families for the collected sequences. Among them, 8 families also appear in the bpRNA-1m data set. Hence, only 481 families with 5265 sequences were suitable and thus retained. These 481 families were also ensured to be in different Rfam clans from sequences in bpRNA-1m. Since most deep-learning-based prediction tools can only handle sequences composed of the four basic nucleotide alphabets, sequences with alphabets other than AU(T)GC were abandoned. For each family, one sequence from each family was selected as the representative. As previously cautioned, benchmark sets with too many short sequences tend to provide biased performance evaluation results.^42^ In order to eliminate the length biases for the remaining bpRNA-new sequences in a family-wise manner, the remaining 481 families are binned with a length of 50 nts, resulting in 10 bins in total (max-length = 489 nts). In these 10 bins, the minimum number of families within a bin is 4. Therefore, to balance the number of sequences in each length bin, we sampled 4 families from each of these 10 bins to prepare the final test set, resulting in 40 test families (i.e., 40 sequences). The test set consisting of these 40 sequences from different families is called TestSetβ in this research. We also confirmed the sequence similarity between the training-validation and test RNA sequences using CD-HIT.^41^ Under the threshold of 0.85, TestSetβ contains no RNA sequence similar to any member of the training-validation set.

#### PDB-Derived Source-Independent Test Set

Since RNA structures deposited in Rfam are actually only a mixture of experimental results, predictions, and homologue modeling inference,^43^ we sought another test set that gathers RNA structures identified with pure experimental evidence. In the bpRNA database,^39^ Danaee et al. also collected some PDB-derived RNA secondary structures that were parsed only from the 3D structures obtained using X-ray crystallography and NMR techniques. We downloaded these PDB-derived results and prepared a source-independent test set named TestSetγ. As in TestSetβ, only sequences represented using AU(T)GC were retained. In addition, to have a fair test set that does not include sequences already used in the training process of any tool, we excluded sequences that have more than 85% CD-HIT-calculated similarity to any sequence from the training-validation set used in this research and the TR1 data set used in the training process of SPOT-RNA. In total, 147 PDB-derived RNA structures were available. We also sought to avoid length biases in TestSetγ by equally length-partitioning these sequences into 10 bins (max-length = 338 nts). We take only 3 sequences within each length bin in order to balance the number of sequences in each bin, resulting in 15 independent sequences (several bins have zero sequence in them) in TestSetγ. Overall, TestSetγ serves as a source-independent test set that helps evaluate the tools in predicting structures directly inferred from experiments.

### Structure Prediction Evaluation Metrics

To evaluate the RNA secondary structure prediction performance, we resorted to the structure F1 score calculation. The precision, recall, and F1 score for a predicted structure are computed as follows^3,8^
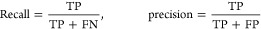
15
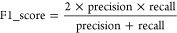
16where TP, FP, and FN stand for the numbers of true-positive, false-positive, and false–negative pairs, respectively. For a predicted structure and its corresponding real structure, TP counts the number of correctly predicted pairings, FP is the number of predicted pairings that do not appear in the real structure, and FN considers the number of real base pairings missed by the prediction. The final F1 score can balance the trade-off between the FP and FN values and is thus used as the overall evaluation metric.

## Results and Discussion

### DEBFold Outperforms Previous Thermodynamics-Based RNA Structure Prediction Tools

We first evaluated the generalization prediction performance of DEBFold and compared its performance with classical thermodynamics-based prediction tools on prepared TestSetα, TestSetβ, and TestSetγ. Nineteen prediction tools that were still publicly available were compared in this section: (1) tools from single-sequence thermodynamics models: RNAfold,^25^ RNALfold,^44^ Fold,^29^ MaxExpect,^26^ RNAprob,^28^ RME (both the PARS-model and the DMS-model),^45^ PKNOTS,^46^ IPknot,^6^ HotKnots,^47^ IterativeHFold,^48^ ShapeKnots,^49^ and ProbKnot;^27^ (2) tools based on homologous sequence alignment and folding: RNAalifold,^14^ TurboFold,^29^ SPARSE,^50^ aliFreeFold,^15^ LocARNA,^51^ comRNA,^52^ and MXSCARNA.^53^ Algorithms that fail to provide executable codes were not included in this comparison. Default parameters suggested by the authors were adopted in these tools for a fair comparison. For IterativeHFold, we set RNAfold as the restriction structure generator for it. For tools that require multiple sequence alignment results, sequences in the same Rfam family were taken. In some Rfam families, the sequences are nearly identical, even though they were collected from different species. These nearly identical sequences within the same family were used in tools that required homologous alignments if no other homologous sequence was available for that family. If no sequence is available in the same RNA structural family of the given sequence, the required homologues were searched against the RNAcentral database^54^ using BLAST as suggested in our previous work.^3^

The results of different tools on TestSetα, TestSetβ, and TestSetγ are summarized in Table 1. In Table 1, the performance of each tool was summarized by its median rank and overall median F1 score on the three test sets. Among single-sequence input tools [RNAfold, RNALfold, Fold, MaxExpect, RNAprob, RME (both the PARS-model and the DMS-model), PKNOTS, IPknot, HotKnots, IterativeHFold, ShapeKnots, and ProbKnot], DEBFold achieves the overall top-one median F1 score rank on the three test sets. The improvement is supposed to be from the integration of various existing thermal knowledge followed by deep learning boosting. Compared with tools that utilize homologous sequences (RNAalifold, TurboFold, SPARSE, aliFreeFold, LocARNA, comRNA, and MXSCARNA), DEBFold still has a better overall median F1 score rank. Combing these comparisons, DEBFold provides cutting-edge performance over existing tools on the test sets. Notice that in TestSetα, tools that rely on sequence alignment results generally perform better than single-sequence input tools since the RNA sequences from Rfam 12.2 mostly come with good family-wise homologous sequences. This advantage may diminish in many real-world use cases, such as in TestSetγ. DEBFold overcomes this restriction by using a deep learning model that integrates the results of many single-sequence structure prediction tools. It is worthwhile noting that DEBFold also boosts the prediction F1 score over the 6 predictions (RNAfold, IPknot, MaxExpect, ProbKnot, RNAprob, and Fold) integrated in the input encoding tensor of the deep network. In summary, DEBFold is concluded to outperform existing classical structure prediction tools with less input requirements.

**Table 1:** Test Set Median F1 Score Performance Comparison between DEBFold and Other Available Thermodynamics-Based RNA Secondary Structure Prediction Tools on the Three Prepared Test Sets^a^

structure prediction tool	TestSetα				TestSetβ				TestSetγ				overall median F1 (%)	median rank
	F1 (%)	P (%)	R (%)	rank	F1 (%)	P (%)	R (%)	rank	F1 (%)	P (%)	R (%)	rank		
DEBFold	64.9	62.1	67.9	4	55.7	56.4	56.8	1	77.9	83.3	73.2	1	64.9	1
RNAalifold	64.7	62.2	67.5	5	55.6	50.7	62.2	2	59.6	56.4	63.3	14	59.6	5
ProbKnot	60.6	60.8	60.7	13	53.8	50.4	58.5	6	72.7	77.8	68.3	6	60.6	6
MaxExpect	64.2	54.9	82.9	7	55.0	43.9	81.7	4	69.2	73.0	65.9	8	64.2	7
ShapeKnots	61.9	54.9	71.8	12	55.2	45.7	69.9	3	70.9	73.7	68.3	7	61.9	7
IterativeHFold	63.5	53.9	82.9	8	52.3	39.9	81.2	8	77.9	83.3	73.2	1	63.5	8
RNAfold	63.5	53.9	82.9	8	52.3	39.9	81.2	8	77.9	83.3	73.2	1	63.5	8
RME (PARS model)	63.5	56.2	75.2	10	54.2	43.1	81.7	5	69.2	73.0	65.9	8	63.5	8
RME (DMS model)	63.1	53.7	82.9	11	53.1	38.8	87.5	7	69.2	73.0	65.9	8	63.1	8
SPARSE	65.7	66.7	64.7	2	51.4	38.9	80.5	10	60.5	72.0	52.2	12	60.5	10
TurboFold	64.6	54.8	83.1	6	47.8	57.1	45.5	13	63.6	85.4	50.7	11	63.6	11
LocARNA	65.7	66.7	64.7	2	49.9	47.8	54.1	11	48.1	53.4	43.7	19	49.9	11
HotKnots	59.1	54.7	75.0	14	48.5	40.7	62.1	12	76.3	82.9	70.7	5	59.1	12
IPknot	57.8	47.0	81.5	15	47.7	44.1	52.0	14	76.9	83.3	66.7	4	57.8	14
aliFreeFold	65.7	59.4	67.1	1	44.9	41.8	49.5	16	48.1	53.4	43.7	19	48.1	16
MXSCARNA	47.2	63.2	37.7	16	34.8	29.8	43.3	19	54.5	100.0	37.5	15	47.2	16
Fold	45.8	36.5	73.5	17	43.2	43.6	42.8	17	54.5	72.7	42.0	15	45.8	17
RNAprob	45.8	36.5	73.5	17	43.2	43.6	42.8	17	54.5	72.7	42.0	15	45.8	17
PKNOTs	42.8	37.7	51.0	20	47.5	42.0	55.6	15	54.2	65.3	46.4	18	47.5	18
RNALfold	45.3	51.9	66.9	19	25.4	31.8	21.1	20	60.5	81.2	48.1	13	45.3	19
comRNA	29.1	24.3	37.8	21									29.1	21

a The median rank column summarizes the rank median of each tool in the three test sets, and the overall median F1 column computes the median F1 score of the three test-set median F1 scores. For comRNA, no reasonable result was provided in TestSetβ and TestSet γ. Notations: P—precision, R—recall.

### DEBFold Has Better Generalization Performance than Existing Deep-Learning-Based Attempts

Recently, more and more deep-learning-based structure prediction tools were developed to help predict RNA secondary structures. We also sought to compare DEBFold to these tools. To the best of our knowledge, existing deep-learning-based RNA secondary structure prediction tools include the following: SPOT-RNA,^21^ SPOT-RNA2,^55^ MXfold2,^22^ GCNFold,^56^ UFold,^57^ REDFold,^58^ and e2eFold.^59^ Since SPOT-RNA and SPOT-RNA2 released by the authors do not encompass any retraining codes and the tools themselves incorporate the whole bpRNA-1m data set in its model training process, independent test sets other than TestSetα, which was prepared from the bpRNA-1m Rfam 12.2 part, should be used to ensure fair comparison between DEBFold and SPOT-RNA/SPOT-RNA2. For this purpose, we downloaded the bpRNA-new data set from the MXfold2 work and prepared TestSetβ based on Rfam 14.4 (excluding those already in Rfam 12.2) for fairly evaluating the performance of DEBFold with existing deep-learning-based prediction tools. In TestSetβ, both the length bias and data contamination issues were eliminated. Details of the TestSetβ preparation steps can be found in the “Contamination-Free Family-Wise Independent Test Set for Evaluating Existing Deep-Learning-Based Structure Prediction Tools” section. Another PDB-derived independent test set, TestSetγ, was also prepared for this purpose. The preparation steps of TestSetγ can be found in the “PDB-Derived Source-Independent Test Set” section.

The final test results of DEBFold and the existing deep-learning-based prediction tools on TestSetα, TestSetβ, and TestSetγ are listed in Table 2. For SPOT-RNA, SPOT-RNA2, and UFold, TestSetα results were not reported due to complete data contamination caused by using the whole bpRNA-1m data set in the training process. REDFold has potential slightly optimistic evaluation results on TestSetα and TestSetβ since a small portion (122 families, whose details were not clearly reported by the original paper) of the Rfam 14.4 families were included in the training process. MXfold2 also included a small portion of the Rfam data (22 Rfam families from Rfam 10.0 and 151 sequences from the S-151Rfam data set, whose details were not clearly reported by the original paper), which might result in potential slightly optimistic results for TestSetα and TestSetβ. We calculated the median performance ranking and median F1 score of each tool on suitable test sets in the comparison. For tools that are suitable to be evaluated on all three test sets (DEBFold, MXfold2, REDFold, GCNfold, and e2efold), DEBFold has superior median F1 score results. Compared with SPOT-RNA, UFold, and SPOT-RNA2, DEBFold shows the top overall median F1 score rank. Among these tools, SPOT-RNA was first pretrained on the bpRNA-1m data set. Then, these pretrained models were fine-tuned and aggregated on the PDB data set. In other words, both the RNA structural families from bpRNA-1m and the PDB-identified RNA sequences were considered in the tool. Because of the final fine-tuning process, the final published SPOT-RNA tool somewhat favors 3D-structure-derived RNA base pairings. However, in PDB-derived TestSetγ, DEBFold still outperforms SPOT-RNA. Therefore, we conclude that DEBFold can provide better results than SPOT-RNA even on the 3D-structure-derived base pairs. It is worth noting that SPOT-RNA2 can achieve good results by considering the homologous sequence information. Nonetheless, SPOT-RNA2 only gets the same median performance rank as DEBFold while demanding more than 7500 times longer execution time than DEBFold (see Table 5). In view of the overall consideration of prediction efficiency and accuracy, DEBFold still has better results than SPOT-RNA2. Overall, the performance improvements in DEBFold are supposed to be attributed to a novel two-stage pipeline that utilizes a 1D structure representation and deep network integration of various thermal models. These analyses indicate that DEBFold can achieve state-of-the-art generalization structure prediction performance over that of currently available deep-learning-based prediction tools.

**Table 2:** Test Set Median F1 Score Performance Comparison between DEBFold and Other Deep-Learning-Based RNA Structure Prediction Tools on the Three Prepared Test Sets^a^

structure prediction tool	TestSetα				TestSetβ				TestSetγ				overall median F1 (%)	median rank
	F1 (%)	P (%)	R (%)	rank	F1 (%)	P (%)	R (%)	rank	F1 (%)	P (%)	R (%)	rank		
**DEBFold**	64.9	62.1	67.9	1	55.7	56.4	56.8	1	77.9	83.3	73.2	2	64.9	1
MXfold2	58.9	62.3	55.9	2	54.3	43.7	73.5	2	78.4	80.6	76.3	1	58.9	2
REDFold	40.4	46.7	37.0	3	41.9	50.6	35.9	3	53.8	56.8	51.2	3	41.9	3
GCNfold	31.9	83.3	20.0	4	27.0	61.0	18.8	4	40.0	81.2	25.0	4	31.9	4
e2eFold	0	0	0	5	1.2	2.3	0.8	5	9.5	26.7	5.8	5	1.2	5
														
**DEBFold**					55.7	56.4	56.8	1	77.9	83.3	73.2	2	66.8	1.5
SPOT-RNA2					51.8	44.0	63.1	2	84.2	84.2	84.2	1	68.0	1.5
SPOT-RNA					50.3	57.1	52.5	3	73.7	93.3	60.9	3	62.0	3
UFold					45.8	33.2	77.1	4	48.3	59.6	40.6	4	47.0	4

a The median rank column summarizes the rank median of each tool in the three test sets, and the overall median F1 column computes the median F1 score of the three test-set median F1 scores. For e2eFold, no reasonable result was provided in TestSetα. Notations: P—precision, R—recall.

### DEBFold Is Robust against Different Thermodynamics-Constrained Optimization Algorithms

The second step of DEBFold feeds the computed structure location folding probabilities into thermodynamically constrained optimization algorithms to obtain the final RNA secondary structure prediction. Previous sections show that DEBFold can have a better structure prediction over the integrated constitutional prediction results by using more accurate folding scores as the optimization soft constraints. We next evaluated the robustness of DEBFold against different constrained optimization algorithms. In DEBFold, Fold was the adopted algorithm for the thermodynamically constrained optimization of the final predicted structures. Besides Fold, RNAfold and ShapeKnots can also help perform thermodynamics-constrained optimization based on soft constraints. These three constrained optimization algorithms were separately combined with DEBFold Stage I (DEBFold-Fold, DEBFold-RNAfold, and DEBFold-ShapeKnots) and then evaluated on TestSetα, TestSetβ, and TestSetγ. The comparison results are summarized in Table 3. As shown in Table 3, DEBFold achieves nearly identical median F1 score results (within one percent) on the three test sets when different thermodynamics-constrained optimization tools are used. From this comparison, it is suggested that DEBFold can provide accurate folding scores that help boost the final structure prediction and is robust against different thermodynamics-constrained optimization tools.

**Table 3:** Performance Evaluation for DEBFold Algorithm Robustness Using the Median F1 Scores on TestSetα, TestSetβ, and TestSetγ^a^

folding algorithm used	TestSetα			TestSetβ			TestSetγ		
	F1 (%)	P (%)	R (%)	F1 (%)	P (%)	R (%)	F1 (%)	P (%)	R (%)
DEBFold-Fold	64.9	62.1	67.9	55.7	56.4	56.8	77.9	83.3	73.2
DEBFold-RNAfold	64.9	62.1	67.9	56.6	54.8	63.7	77.9	83.3	73.2
DEBFold-ShapeKnots	64.9	62.1	67.9	55.5	55.7	56.8	77.9	83.3	73.2

a DEBFold Stage I deep network combined with different thermodynamics-constrained optimization algorithms were compared. Notations: P—precision, R—recall.

### DEBFold Provides Superior Structure Location Labeling Results to the Baseline and the Existing Model

In DEBFold Stage 1, the base locations where the structure pairings occur are labeled. The higher performance of the labeling process usually leads to better constrained optimization results. We compared the structure location labeling performance of DEBFold Stage 1 with the simple consensus model and another existing technique called GRASP.^60^ In DEBFold Stage 1, the probability of a base location being involved in the final structure pairings is predicted by a deep convolutional network. In previous research, Ke et al. proposed using the XGBoost model on the flattened one-hot encoding tensor of a sequence window to get the pairing probability of an RNA base location and implemented this method as a tool called GRASP.^60^ In both DEBFold Stage 1 and GRASP, a fair threshold of 0.5 was adopted when the existence of base pairings was labeled for different RNA base locations. On the other hand, the simple consensus model labels the existence of the base pairing for an RNA base location if all 6 tools (RNAfold,^25^ IPknot,^6^ MaxExpect,^26^ ProbKnot,^27^ RNAProb,^28^ and Fold^29^) agree on the pairing existence. In order to evaluate the location labeling results, we resort to the confusion matrix generated from the labeling results of a specific tool for each RNA. For structure location labeling in each RNA, TP is the number of correctly labeled pairing locations, FN represents the number of missed pairing locations, FP shows the number of nonpairing locations mistakenly marked to be paired, and TN counts the number of correctly labeled nonpairing locations. Based on the defined confusion matrix, the recall, precision, and F1 score values for the structure location labeling problem are defined similarly to those in eqs 15 and 16. We calculated the evaluation metrics for each RNA on the three prepared test sets (TestSetα, TestSetβ, and TestSetγ) and obtained the median F1 score of all of the RNAs in each test set. As summarized in Table 4, DEBFold Stage 1 achieves F1 scores superior to those of both the consensus model and GRASP on all three test sets. By this comparison, we conclude that DEBFold Stage 1 is better than the simple consensus model and the existing tool GRASP in labeling the existence of the base pairings for RNA base locations.

**Table 4:** Labeling Performance Comparison among DEBFold, GRASP, and the Simple Consensus Model on the Three Test Sets (TestSetα, TestSetβ, and TestSetγ)^a^

folding algorithm used	TestSetα			TestSetβ			TestSetγ		
	F1 (%)	P (%)	R (%)	F1 (%)	P (%)	R (%)	F1 (%)	P (%)	R (%)
**DEBFold Stage 1**	77.0	72.1	82.5	71.8	61.8	85.5	79.5	87.0	73.2
the simple consensus model	70.7	76.3	65.6	55.9	63.3	50.0	77.2	88.9	68.3
GRASP	52.5	45.7	61.5	51.0	48.1	54.2	63.3	77.6	53.5

a The median value is recorded for each test set. Notations: P – precision, R – recall.

### Speed Comparison of DEBFold with the Existing Tools

Due to the advancement of sequencing technology, lots of RNA sequences are found, leading to the need for large-scale structure investigation directly on the sequences. To fulfill this need, the execution time required for each prediction tool should also be inspected in addition to structure prediction accuracy. We evaluated and compared the execution time of DEBFold and different structure prediction tools on the collected 147 PDB-derived sequences using a workstation with Intel i7 CPU cores and 128GB RAM. The results are summarized in Table 5. On these 147 RNA sequences, most single-input folding algorithms used less than a minute to process the predictions. Since DEBFold integrates the outcomes of six different single-input folding prediction results, it took around 1 min to finish all predictions. For algorithms that consider homologous sequences (RNAalifold,^14^ TurboFold,^29^ SPARSE,^50^ aliFreeFold,^15^ LocARNA,^51^ comRNA,^52^ and MXSCARNA^53^), around an additional 10 min was demanded to get the homologous sequences using BLAST. Therefore, DEBFold has better time efficiency than these homology-based tools and achieves better performance. In deep-learning-based predictions, SPOT-RNA required about 3 times more execution time than did DEBFold. Among all tools, SPOT-RNA2 had very low execution efficiency and needed more than 142 h, which is more than 7500 times as long as the execution time of DEBFold, to predict merely the structures of the 15 TestSetγ sequences selected by stratified sampling from all 147 RNAs. More execution time is required to finish all 147 RNAs. The high execution time of SPOT-RNA2 suggests that SPOT-RNA2 is probably not ready for a large-scale RNA structure investigation. Moreover, SPOT-RNA2 requires more than 2TB of disk space to store the information needed for its algorithm. In summary, DEBFold not only achieves state-of-the-art prediction accuracy but also retains an acceptable execution time for large-scale RNA sequence investigation.

**Table 5:** Execution Time Comparison between DEBFold and Other Available RNA Secondary Structure Prediction Tools on the Collected 147 PDB-Derived Sequences

structure prediction tool	execution time (s)
**DEBFold**	67.3
RNAfold	7.7
RNALfold	9.2
PKNOTS	12.2
Fold	13.4
IterativeHFold	15.5
IPknot	17.3
ProbKnot	20.5
MaxExpect	20.6
RNAProb	22.1
ShapeKnots	382.8
HotKnots	2824.0
RME (PARS model + DMS model)	74906.6
(Homology finding using BLAST)	591.1
RNAalifold	6.1
SPARSE	10.1
MXSCARNA	14.9
LocARNA	22.9
aliFreeFold	31.6
TurboFold	190.0
comRNA	904.3
MXfold2	33.6
GCNfold	63.8
REDFold	72.9
UFold	102.8
e2eFold	123.5
SPOT-RNA	192.8
SPOT-RNA2	511970.3^a^

a Note that the execution time accumulated for SPOT-RNA2 is only from the prediction of the 15 TestSetγ sequences selected by stratified sampling from the total available 147 PDB-derived sequences.

### Limitations of DEBFold

In this work, we developed a deep-learning model that can integrate the prediction results from different single-sequence folding tools to more accurately predict RNA secondary structures. DEBFold is a two-stage pipeline for RNA secondary structure prediction. In the first stage, DEBFold identifies the locations where structural pairings occur. Then, the pairing probabilities for the base locations of a given sequence are transformed into the SHAPE-like score tensor. Based on the SHAPE-like score tensor, the structure prediction bearing the minimum free energy under these soft constraints is given by Fold. In RNA structure prediction, pseudoknots (intertwined helices on a plane) and noncanonical pairings (the base pairs formed by hydrogen bonding differing from the patterns of standard Watson–Crick rules^61^) present significant challenges for prediction algorithms. Since DEBFold Stage 1 does not distinguish pseudoknot, canonical, and noncanonical RNA pairings, these pairings are all potentially considered in this stage. It is possible to adopt ShapeKnots or other minimum free energy structure prediction optimizers in DEBFold Stage 2 if these challenging types of base pairings are to be considered in the final optimization process. However, since there are insufficient noncanonical or pseudoknot pairings in the ground-truth data set, DEBFold currently mainly focuses on canonical base pairings.

Previously, Szikszai et al.^23^ pointed out that deep-learning models for RNA secondary structure prediction can easily overfit a data set if a sequence-based cross-validation and a test process are adopted. This type of overfitting occurs even when a massive number of sequences from only a few families are provided. Szikszai et al. argued that the chief cause for the phenomenon lies within the structural similarities among RNA sequences in the same family. Based on their research, we adopted a family-wise approach in developing DEBFold and further prepared test sets that are more suitable for evaluating this problem in existing deep-learning-based prediction tools. Although DEBFold overcomes the overfitting problem and provides state-of-the-art performance, inherent limitations remain. Currently, the known RNA families are far smaller than the known sequences in the community. Despite the large number of RNAs with known sequences available, most of them belong to the same structural families. Because of the limited RNA structural families available (only 2606 different families in Rfam 14.4), the performance of single-sequence structure prediction using deep learning is largely restricted. The limited RNA structural families also constrain the prediction accuracy of long noncoding RNAs (lncRNAs) since the overfitting problem boils down to the lack of structural family diversity. Currently, popular data sets contain scarce numbers of structural families for long RNAs, inhibiting the structural prediction of lncRNAs. The current version of DEBFold is suitable only for sequences up to 512 bps. Although some of the existing deep-learning-based tools claim to be able to deal with lncRNAs, the moderate performance observed in Table 2 indicates that the originally claimed prediction accuracy may still need more attention. Notice that DEBFold performs better than classical folding algorithms that incorporate multiple-sequence alignments. Therefore, whether multiple-sequence alignments can better help suggest structure modules in the DEBFold pipeline requires further study.

## Conclusions

In this research, we designed and implemented a deep learning tool called DEBFold to provide accurate RNA secondary structure prediction for sequences across RNA structural families. DEBFold is verified to be free of the overfitting pitfall occurring in many deep-learning-based structure prediction tools and outperforms the currently available tools. We believe that the development of DEBFold can significantly accelerate the use of artificial intelligence to help us understand structure–function relations for RNAs.

## Data Availability

The implemented DEBFold pipeline and the processed RNA structure ground-truth data sets (including the training-validation set, TestSetα, TestSetβ, and TestSetγ) are available at https://cobis.bme.ncku.edu.tw/DEBFold and https://github.com/cobisLab/DEBFold.
